# Endometriosis of the Small Bowel: A Diagnostic Enigma

**DOI:** 10.7759/cureus.15520

**Published:** 2021-06-08

**Authors:** Saqib Mehmood, Sarah Zhao, Quratul Ain, Jonathan Van Dellen, Caitlin Beggan

**Affiliations:** 1 General Surgery, Croydon University Hospital, London, GBR; 2 Pathology, St George’s University Hospital, London, GBR

**Keywords:** endometriosis, extrauterine spread, meckel’s diverticulum, intestinal endometriosis, diagnostic laparoscopy

## Abstract

Endometriosis refers to the implantation and proliferation of endometrial tissue outside the uterus. Small bowel endometriosis is an uncommon location for endometrial deposits and when present, it can pose diagnostic difficulty. Here, we present a case of a 50-year-old female with small bowel endometriosis who suffered from recurrent attacks of colicky abdominal pain for few months. Her cross-sectional investigations remained largely inconclusive. Ultimately, she underwent diagnostic laparoscopy which was diagnostic and therapeutic.

## Introduction

Endometriosis is an important cause of chronic abdominal pain in women of childbearing age. It refers to the implantation and proliferation of functional endometrial tissue outside the uterus [1]. Reported prevalence ranges from 6% to 10% in women of reproductive age. The majority of patients experience chronic abdominal pain, while others may experience abnormal menstruation or infertility. Endometrial deposits can be found in various locations such as the peritoneum, rectovaginal septum, and ovaries. However, rarely it may involve the pericardium, pleura, small and large intestine, and other organs [2].

Bowel endometriosis may be entirely asymptomatic. However, it may cause a constellation of symptoms such as nausea, vomiting, recurrent colicky abdominal pain, rectal pain, mass formation, and rarely intestinal obstruction [3]. Recto sigmoid is the most common intestinal site of endometriosis, accounting for 50-90% of the cases, followed by small bowel, appendix, and cecum accounting for 2-16%, 3-18%, and 2-5% of the cases, respectively [4].

In this report, we are presenting a case of symptomatic ileal endometriosis necessitating bowel resection. The patient underwent multiple investigations without a definitive diagnosis. She eventually underwent diagnostic laparoscopy. Surgical findings were consistent with endometriosis of the small bowel which was later confirmed on histology. This report serves as a reminder that small bowel endometriosis is a significant cause of chronic abdominal pain which may initially be overlooked. Therefore, when present, it may present a unique diagnostic challenge.

## Case presentation

A 50-year-old female was referred to a surgical clinic with troublesome recurrent abdominal pain for few months, few episodes of which had culminated in acute hospital admission. The pain was spasmodic in nature and associated with vomiting. She also described rectal bleeding and diarrhoea on a few occasions. Her history included a laparoscopy for diagnosis and treatment of pelvic endometriosis. She also underwent endometrial ablation for menorrhagia a few years ago.

Her abdomen was soft and non-tender, with no obvious masses or organomegaly. Her lab workup did not show any abnormality, she had normal hemoglobin (Hb) of 136 g/L (115-165 g/L), urea 5.5 mmol/L (2.5-7.8 mmol/L), and creatinine 65 µmol/L (45-84 µmol/L). Her faecal analysis was also unremarkable. She underwent a contrast-enhanced CT scan abdomen/pelvis, which did not show any abnormality. Also, a full colonoscopy was carried out, which did not show any anomaly.

**Figure 1:**
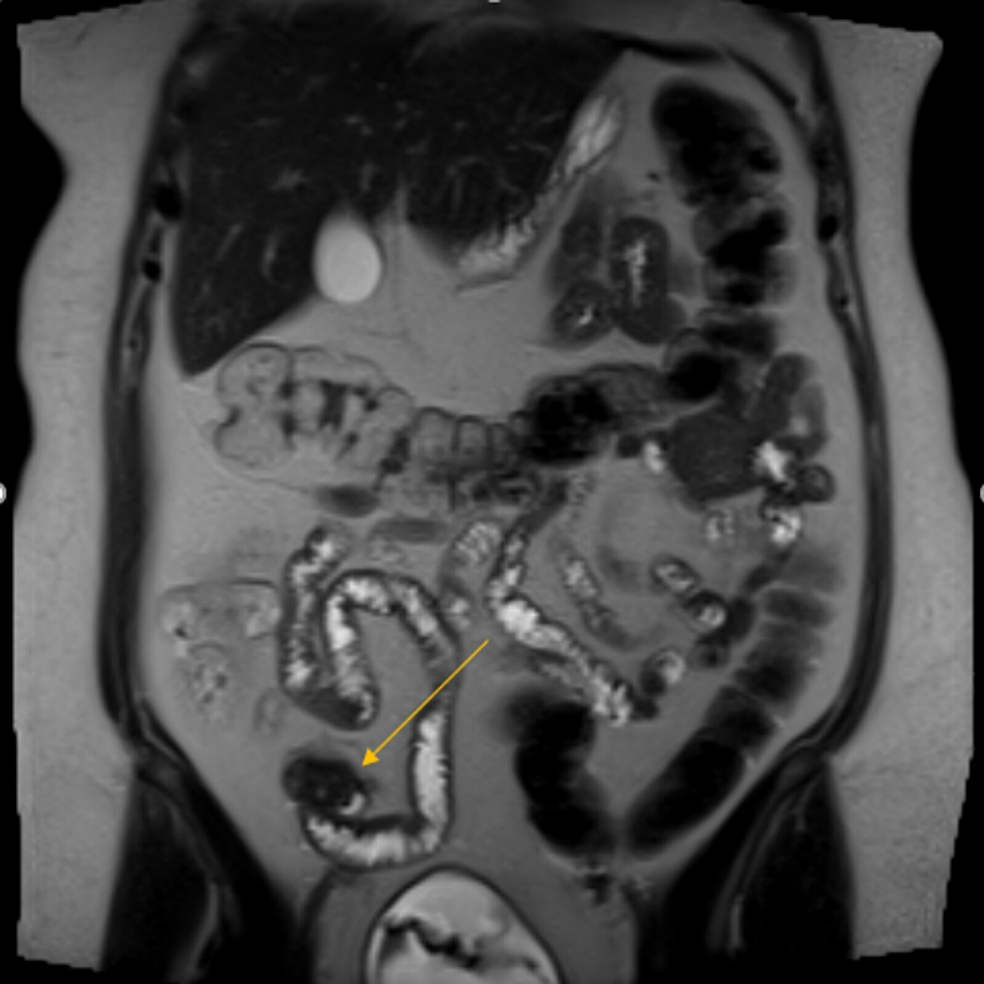
MRI abdomen T2 HASTE coronal view: image demonstrates an area of abnormal peri-enteric thickening in the distal ileum in the right iliac fossa. Differentials included Meckel's diverticulum and endometriosis of the small bowel.

Her MRI abdomen demonstrated an area of abnormal perienteric thickening in the distal ileum raising the possibility of either a Meckel's diverticulum or endometrial deposit. It was followed up by a small bowel MRI which remained equivocal, however, suggested that if thickened small bowel was just a tortuous small bowel loop (Figure 1).

**Figure 2:**
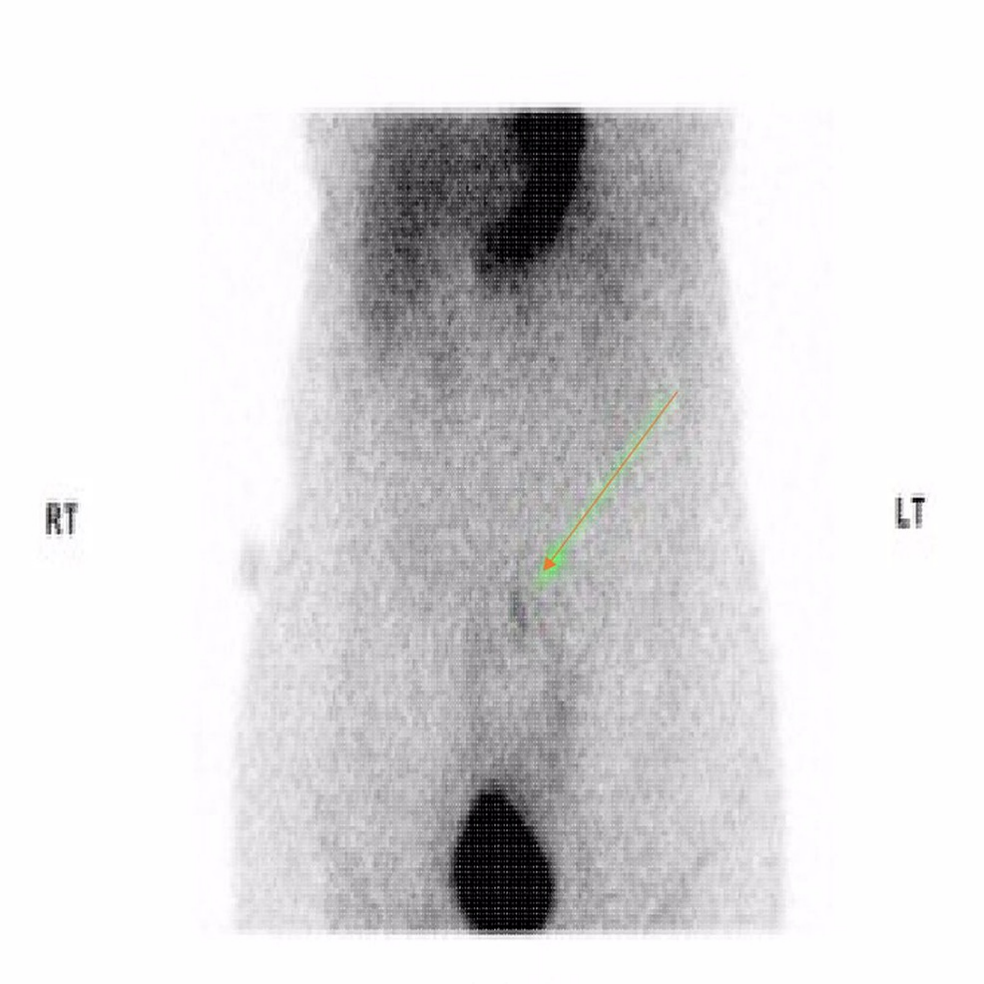
Technetium-99m (99mTc) nuclear medicine Meckel's scan: showing an area of tracer uptake in mid to distal ileum suggesting the possibility of Meckel's diverticulum.

The patient also underwent a Technetium-99m (99mTc) nuclear medicine Meckel's scan, which interestingly showed an area of tracer uptake and suggested the possibility of Meckel's diverticulum (Figure 2).

**Figure 3:**
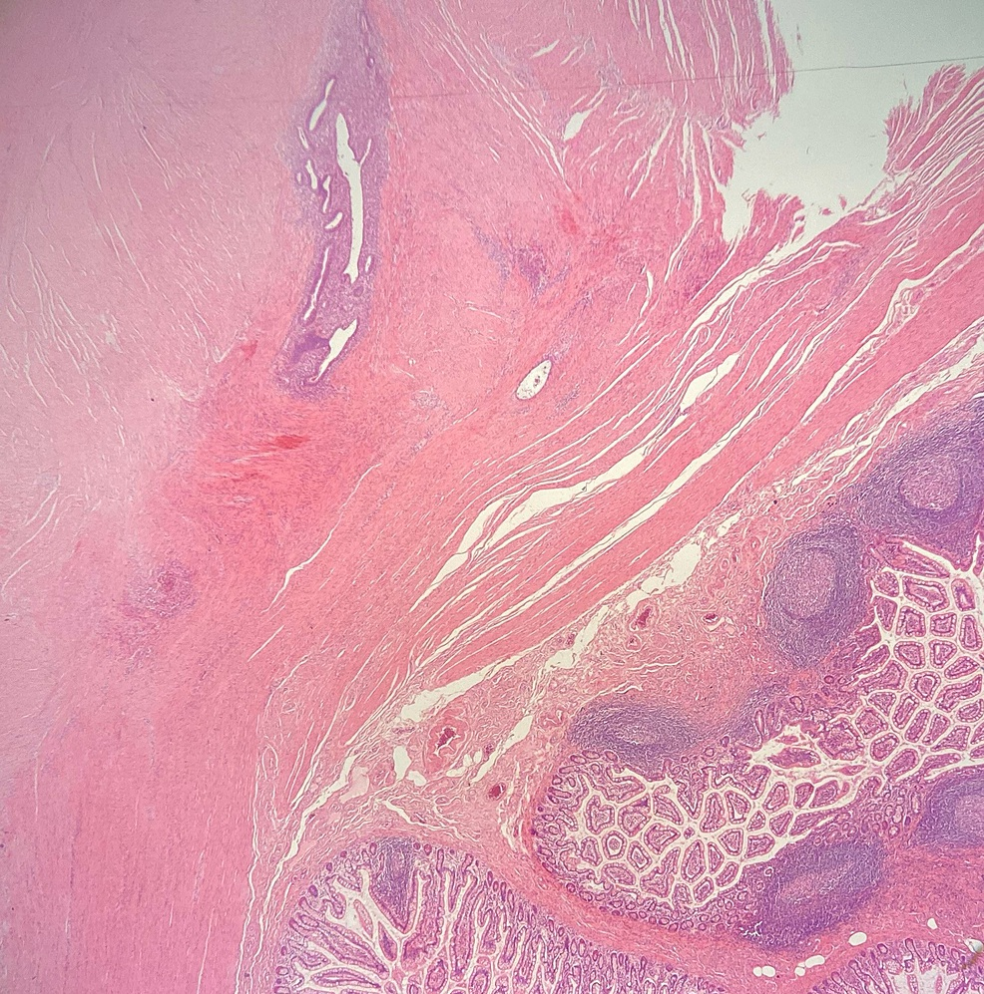
Photomicrograph of haematoxylin and eosin-stained section (×20 magnification) showing endometrial glands and stroma within thick smooth muscle bundles of muscularis propria. Overlying small bowel mucosa is normal.

After a complete set of investigations, the patient underwent diagnostic laparoscopy, which demonstrated a distal small bowel mesenteric lesion causing kinking of the small bowel. Upon release, a chocolate-colored fluid was noted from the lesion. The involved small bowel was resected, and a side to side anastomosis was fashioned. The small bowel was thoroughly looked for the presence of Meckel's diverticulum, which was not present. Histology results were consistent with an endometrial deposit of the small bowel, and no other abnormality was found (Figure 3).

Post-operatively, the patient has made a good recovery and remains symptoms-free to date.

## Discussion

The exact aetiology of endometriosis remains unclear. However, certain theories have been proposed to elucidate their origin. One such theory is Sampsons' theory of retrograde menstruation: which suggests that during menstruation, endometrial tissue emigrates through the fallopian tube to form serosal deposits on abdominal and pelvic organs [5,6]. Alternatively, Minh's theory proposes that extrauterine endometrial tissue could result from the metaplastic transformation of pluripotent peritoneal mesothelium [7]. Extrauterine deposits of endometrial tissue have also been attributed to lymphatic and hematogenous spread [8]. Implantation and proliferation of endometrial tissue at ectopic sites are thought to be facilitated by neo-vascularization arbitrated by pro-angiogenic factors, e.g., vascular endothelial growth factor [9].

Endometrial tissue, regardless of its location, is hormone-responsive and can proliferate to form cysts and nodules, which can cause chronic pain, bleeding, and scarring [8-10]. The intestinal endometriosis symptoms range from nausea, vomiting, abdominal pain, abdominal distension, tenesmus, painful defecation, and rectal bleeding, counting on the location of involvement [11]. It is most commonly found in the recto-sigmoid region, followed by the small bowel, appendix, and cecum [4].

Small bowel endometriosis is thought to affect serosa, and deposits tend to be less than 2 cm. In most cases, it is asymptomatic; however, it may lead to chronic inflammation leading to fibrosis, which successively accounts for most of the gastrointestinal tract symptoms. However, the transmural spread has also been observed, resulting in rectal bleeding, bowel obstruction, and mass formation mimicking tumors [12]. Rarely, it may lead to intussusception, malignant transformation, and intestinal perforation [12,13].

It can be challenging to diagnose bowel endometriosis in a timely and precise manner, as was the case with our patient. However, bowel endometriosis is to be suspected in otherwise fit and well young female patients, especially when there is a history of concomitant pelvic endometriosis.

CT scan can help identify a thickened, stenosing mass; however, it is rarely diagnostic of endometrial deposits. A contrast-enhanced CT scan in our patient did not demonstrate any abnormality in the small bowel. Multislice CT, however, when combined with enteroclysis (MSCTe), has a high potential for detecting alterations in the intestinal wall. In a study of 98 women with symptoms indicative of colorectal endometriosis, Biscaldi et al. found that MSCTe reported 94.8% of bowel endometriotic nodules [14].

Since endometrial tissue involves the deeper layers of the abdominal wall, endoscopic biopsies may yield insufficient information required for diagnosis. When bowel mucosa is involved with endometriosis, it may result in a non-specific pattern of inflammation; therefore, it can be challenging to differentiate it from other forms of bowel disease such as ischemic colitis or inflammatory bowel disease [15].

Magnetic resonance imaging has high sensitivity in diagnosing endometriosis (77-93%). MRI has also been concomitantly used with rectal ultrasound. Sensitivity and negative predictive value of rectal ultrasound range from 92% to 100%. However, it has poor specificity and positive predictive values, 66% and 83-94% [16,17].

In our patient, MRI raised a possibility of thickened bowel loop in distal small bowel suggestive of either a Meckel's diverticulum or an endometrial deposit. It was followed up by a nuclear medicine Meckel's scan, which showed tracer uptake at the level of mid to distal ileum suggestive of Meckel's diverticulum.

Diagnostic laparoscopy and biopsy remain the gold standard treatment in suspected endometrial deposits of the bowel wall. It allows visualization of abdominal and pelvic organs and allows resection if required [18]. In the case under discussion, diagnostic laparoscopy was diagnostic and therapeutic.

There are surgical and medical approaches to the treatment of endometriosis. The surgical approach allows the correct diagnosis and treatment at the same time in cases where a diagnosis has not been obtained with confidence pre-operatively. Whereas a medical approach can be utilized to treat confirmed cases of bowel endometriosis which are not causing obstructive symptoms. Few drugs have been proven to reduce the size and symptoms of endometriosis effectively; these include nonsteroidal anti-inflammatory drugs (NSAIDs) combined with oral contraceptives, aromatase inhibitors, and gonadotropin-releasing hormone agonists. These drugs function by reducing inflammation, suppressing or interrupting ovarian hormone development, resulting in hypoestrogenic conditions and endometrial atrophy [19,20].

The severity and clinical appearance of intestinal endometriosis determine the surgical approach. Patients with small deposits can be offered medical treatment first, with elective resection scheduled later. On the other hand, resection of the affected bowel is needed for large lesions causing an imminent obstruction. Surgical management was utilized as a diagnostic and therapeutic approach in our patients.

## Conclusions

Even though various imaging modalities are readily available to aid in the diagnosis of bowel endometriosis, it can still be challenging to diagnose it and be mistaken for other diseases. The possibility of intestinal endometriosis should be carefully considered, particularly in young female patients with a history of endometriosis elsewhere in the body. Laparoscopy remains the gold standard diagnostic and therapeutic approach.
